# Star players sidelined in chloride homeostasis in neurons

**DOI:** 10.3389/fncel.2014.00114

**Published:** 2014-04-24

**Authors:** Chang-Hoon Cho

**Affiliations:** Graduate School of Life Science, Institute of Biotechnology, Korea UniversitySeoul, Korea

**Keywords:** chloride homeostasis, KCC2, NKCC1, impermeant anions, Clomeleon, extracellular matrix

The chloride ion (Cl^−^) is the most abundant physiological anion and is involved in many cellular functions including intracellular membrane trafficking, volume control, and excitability, as well regulating short- and long-term plasticity in neurons (Woodin et al., 2003; De Koninck, 2007; Raimondo et al., 2012; Succol et al., 2012; Stauber and Jentsch, 2013). The intracellular chloride concentration ([Cl^−^]_i_) is regulated by various chloride-permeable transporters and ion channels. Mutations and/or malfunction of Cl^−^-permeable membrane proteins may disturb chloride homeostasis leading to diverse disease states including hypertension, hepatic encephalopathy, neuropathic pain, and epilepsy (Huberfeld et al., 2007; Price et al., 2009; Li et al., 2012; Ye et al., 2012).

The neurotransmitter GABA (γ-aminobutyric acid) binds to a family of pentameric, ligand-gated Cl^−^ channels (GABA_A_ Receptors). In adult neurons, GABA is principally inhibitory, while in immature neurons, GABA can be excitatory. These pleiotropic effects of GABA are believed to be controlled by [Cl^−^]_i_, which is developmentally regulated by two cation chloride cotransporters (CCCs), NKCC1, and KCC2, that allow Cl^−^ to move in and out of the cells, respectively (Ben-Ari et al., 2007; Blaesse et al., 2009). Although the established excitatory GABA hypothesis has been challenged over the years (most recently by Bregestovski and Bernard, 2012), there is no broadly accepted alternative mechanism for regulating [Cl^−^]_i_ without the major involvement of these two CCCs.

Subcellular [Cl^−^]_i_ is not homogenously distributed within cells. It is generally thought that differential subcellular expressions of CCCs, other Cl^−^ permeable membrane proteins and region-specific subcellular structures may contribute to the creation of microdomains of [Cl^−^]_i_ (Gavrikov et al., 2006; Szabadics et al., 2006; El-Hassar et al., 2007; Khirug et al., 2008; Báldi et al., 2010). Traditionally, the perforated-patch recording technique (e.g., gramicidin) has been used to monitor [Cl^−^]_i_ without disrupting the intracellular environment (Ebihara et al., 1995). Fluorescent Cl^−^ chemical indicators (e.g., MQAE) and FRET (Fluorescence resonance energy transfer)-based ratiometric Cl^−^ sensors (e.g., Clomeleon and ClopHensor) have been developed and used (Verkman et al., 1989; Kuner and Augustine, 2000; Arosio et al., 2010).

Using a genetic indicator of [Cl^−^]/pH (Clomeleon), Dr. Kevin Staley's group at Harvard Medical School reported a new mechanism for establishing [Cl^−^]_i_ in neurons (Glykys et al., 2014). In summary, by measuring fluorescence changes based on the concentration gradient of Cl^−^ across the cell membrane, they hypothesized that the balance between cytoplasmic impermeant anions (e.g., negatively charged DNA and proteins at physiological pH) and polyanonic extracellular matrix glycoproteins (e.g., sulfates on proteoglycans) constrains the local [Cl^−^]_i_ in acute and cultured brain slices from Clomeleon mice. They observed a small difference in [Cl^−^]_i_ between two developmentally different ages (P8–P9 and P32–44) that previously were thought to exhibit large differences in [Cl^−^]_i_ (Ben-Ari et al., 2007; Blaesse et al., 2009). They treated acute hippocampal and neocortical slices with a KCC2 inhibitor (VU0240551), or an NKCC1 inhibitor (bumetanide) at two different ages. However, these two antagonists did not show much effect in altering [Cl^−^]_i_. They also used Alcian blue to stain extracellular sulfated glycosaminoglycans and SYTO64 to label cytoplasmic-nuclear nucleic acids and found a negative correlation between staining density and [Cl^−^]_i_. They have observed an increase in [Cl^−^]_i_ by treating organotypic hippocampal slices with chondroitinase ABC to release SO^−^_4_ from the extracellular matrix (ECM) as well. They concluded that local [Cl^−^]_i_ is at equilibrium at different local [anion], and CCCs are not required to compensate for intracytoplasmic Cl^−^ diffusion. This is surprising.

The interpretation of these results must proceed with caution. While Clomeleon permits non-invasive monitoring activity, its affinity for Cl^−^ is well beyond the physiological range of [Cl^−^] (Kuner and Augustine, 2000; Berglund et al., 2006). There are also serious issues with this study. First, viability of brain slices: simply obtaining field recordings could have been a good indication of the condition of cells. However, the only measurement of this was an apoptosis assay after experiments with chondroitinase. Second, taking advantage of Clomeleon for the multicellular imaging is an informative approach especially when cell populations are heterogeneous in [Cl^−^]_i_. However, the imaging scanning speed (which was not mentioned) must be at least hundreds times slower than any physiological change which can be easily assessed by conventional electrophysiological recordings. Therefore, it is a desired practice to combine two techniques—at least sequentially unless simultaneously possible—which this group failed to do, except for showing epileptiform activities in the organotypic slice cultures. It is possible to speculate that spending enough time to do “z-stack” scanning for this study allows for dynamic chloride movement to be largely missed. The third issue is the choice of the filter to separate YFP from CFP signals. This group used a bandpass filter for yellow (500–540 nm) to acquire the fluorescent emission. However, emissions could have been split at 515 nm to minimize the signal contamination between two fluorescent proteins like the way it was used in the original Clomeleon paper (Kuner and Augustine, 2000). Therefore, the signal detection seems to be less reliable in the paper. The fourth issue is bumetanide: in similar experiments, perforated patch recordings have been used to show that bumetanide is an effective NKCC1 inhibitor (e.g., Sipilä et al., 2006; Lagostena et al., 2010). Even this group has reported the same in a near identical experiment (Brumback and Staley, 2008). How do they explain these two different results? Finally, in a previous report this group claimed that brain slicing itself causes severe damages to the surface of the slices. Therefore, to measure [Cl^−^]_i_ of undamaged cells, you need to look 200 μm deep into the brain slices (Dzhala et al., 2012). This group did not describe the depth of the plane in the slices they have imaged and maybe that's why they observed a wide range of [Cl^−^]_i_. Having that much [Cl^−^]_i_ could place you out of the range of physiological reversal potential values for GABA (Berglund et al., 2006).

Because of these and other troubling issues in this paper, many will have reservations about sidelining two star players from regulating intracellular chloride homeostasis.

## Conflict of interest statement

The author declares that the research was conducted in the absence of any commercial or financial relationships that could be construed as a potential conflict of interest.
